# The MLR, NLR, PLR and D-dimer are associated with clinical outcome in lung cancer patients treated with surgery

**DOI:** 10.1186/s12890-022-01901-7

**Published:** 2022-03-25

**Authors:** Jun Wang, Huawei li, Ran Xu, Tong Lu, Jiaying Zhao, Pengfei Zhang, Lidong Qu, Shengqiang Zhang, Jida Guo, Linyou Zhang

**Affiliations:** Department of Thoracic Surgery, The Second Affiliated Hospital of Harbin Medical University, Harbin, China

**Keywords:** Peripheral blood biomarkers, Early lung cancer, MLR, NLR, PLR, D-dimer

## Abstract

**Objective:**

The study objective was to investigate the use of peripheral blood biomarkers as predictors of patient survival. The aim of this study was to identify the baseline peripheral blood biomarkers associated with clinical outcome in patients with early lung cancer (stage I-II) treated with surgery.

**Methods:**

We included and analysed data from 376 patients with early-stage lung cancer who underwent a standard lobectomy. Univariate and multivariate Cox regression analyses were performed on all patients to assess the relationships between progression-free survival (PFS) and overall survival (OS) and the peripheral blood biomarker metrics measured before surgical treatment. The peripheral blood parameters included monocyte-to-lymphocyte ratio (MLR), neutrophil-to-lymphocyte ratio (NLR), platelet-to-lymphocyte ratio (PLR) and dimeric fibrin fragment D (D-dimer).

**Results:**

After univariate Cox regression analysis, low MLR, low NLR, low PLR and low D-dimer values were significantly associated with both better OS and PFS (all *p* < 0.05). In multivariate Cox regression analysis, a low MLR was significantly and independently associated with both better overall survival and better progression-free survival (both *p* <0.05). A low D-dimer level was significantly and independently associated with better overall survival (*p* <0.05). Furthermore, the categorization of patients according to the number of factors with favourable results revealed that those without favourable results had significantly worse outcomes than that of those patients with at least one.

**Conclusion:**

A baseline signature of low MLR, low NLR, low PLR, and low D-dimer values was associated with a better survival outcome for patients treated with surgery. Patients with more favourable results had better survival outcomes.

**Supplementary Information:**

The online version contains supplementary material available at 10.1186/s12890-022-01901-7.

## Background

Surgery for early-stage lung cancer is an indisputable necessity. However, even when patients undergo the same standard lobectomy, a large difference in patient survival times has been demonstrated. Therefore, we need to find a preoperative blood biomarker to effectively identify those surgical patients with poor prognosis to carry out effective intervention to prolong their lives.

In recent years, many studies have demonstrated the important role of peripheral blood markers in the prognosis of patients with various tumours. The NLR might predict clinical responses to eribulin-based treatment in patients with metastatic breast cancer ^6^. In patients with HER2 + breast cancer, a lower NLR and lower MLR indicate a longer survival [1, 2]. It was reported that plasma D-dimer was regarded as a prognostic marker for various types of malignancies, including non-small-cell lung carcinoma (NSCLC) [3]. In addition, peripheral blood biomarkers have shown good prognostic ability in a variety of solid tumours, including melanoma [4], colorectal cancer [5], oesophageal cancer [6] and pancreatic cancer [7]. The above studies strongly suggest that single peripheral blood biomarkers have good prognostic abilities in patients with malignancies who have undergone different treatments. However, whether the peripheral blood parameters of patients with early-stage lung cancer treated by surgery are related to their survival outcomes needs to be further elucidated.

## Methods

### Patients

This study retrospectively analysed the medical records of 376 patients with stage I-II LC at the Department of Thoracic Surgery of the Second Affiliated Hospital of Harbin Medical University in Heilongjiang Province, China, from January 2015 to July 2017. All patients received standard lobectomy, and 84 patients received postoperative adjuvant therapy, including chemotherapy, immunotherapy, and targeted therapy. Inclusion criteria were as follows: (1) preoperative imaging suggestive of a mass confined to a single lung lobe; (2) no distant metastases; (3) lack of preoperative adjuvant medication; (4) lack of haematological malignancies; (5) complete clinical and follow-up information; and (6) survival for at least 30 days postoperatively. Patients were followed up every three months after surgery via outpatient clinics or over the phone, with the last follow-up visit for all patients performed on June 30, 2020.

### Data collection

No patients underwent preoperative biopsy, and only intraoperative and postoperative biopsies were performed. All LC patients were newly diagnosed and classified by clinical symptoms and pathological detection according to International Association for the Study of Lung Cancer (IASLC) TNM staging[8]. Finally, patients with TNM stage I-II were selected. Peripheral blood biomarkers levels, including neutrophil count (10^9/L), monocyte count (10^9/L), lymphocyte count (10^9/L), platelet count (10^9/L) and D-dimer concentration (ng/ml), were collected from the electronic medical record within 3 days prior to the procedure, as well as the patient's age, sex, BMI, underlying disease history, pathology profile, ECOG score and other basic clinical information. The ratios of the WBC counts were calculated as follows: NLR = number of neutrophils divided by number lymphocytes, MLR = number of monocytes divided by number of lymphocytes and PLR = platelet count divided by number of lymphocytes. Survival rates were analysed by PFS and OS.

### Follow-up

After surgery therapy, all the patients were followed up every 3 months to obtain survival data via outpatient clinics or over the phone. Each patient underwent at least 6 CT imaging examinations during the follow-up period to collect survival data into medical records. The 3-year OS was the determined endpoint in our study, and 30 June 2020, was the deadline of the follow‐up. OS was calculated from the date of diagnosis to the date of death from any cause or at the date of the last follow-up interview. PFS was calculated from the date of diagnosis to the date of disease progression, relapse, or death from any cause, whichever came first.

### Statistical analysis

Receiver operating characteristic (ROC) curves were generated to search for the best cut-off values for MLR, NLR, PLR and D-dimer to stratify patients at a high risk of death. In this ROC curve, the point with the highest Youden index (defined as sensitivity + specificity -1) was selected as the best cut-off value (Additional file 1). This study used SPSS version 25.0 (IBM Corp, Armonk, NY, USA) and R version 4.1.0 (2021–05-18) for analysis of baseline statistics of patient clinical data. Moreover, progression-free survival (PFS) and overall survival (OS) were calculated by the Kaplan–Meier method, while the log-rank test was used for comparison. Univariate and multivariate Cox regression were used to determine the 95% confidence interval (CI) and risk ratio (HR). Parameters with a p value less than 0.05 in the univariable analysis were selected for inclusion in multivariable analysis. Clinically important factors included sex, smoking, pathology, pleural invasion status (PIS) and Eastern Cooperative Oncology Group performance status (ECOG PS). *p* <0.05 in two-sided tests was considered statistically significant.

## Result

### Patient demographics and clinical characteristics

As shown in Table 1. 376 patients with early LC who underwent surgery were ultimately identified. The results showed that there were 218 cases (58%) in males and 158 cases (42%) in females. The median age was 60 (53,65) years. In our sample, the majority of patients had an ECOG physical status of 0–1 (320/376, 85.1%), and the majority of patients had lung adenocarcinoma (n = 233/376, 62%). The median follow-up times for PFS and OS in our study were 1090.5 days and 1110 days, respectively. During the median follow-up time, 107 patients progressed, and 94 patients died (Additional file 2, Table 5).

**Table 1:** Patients’ characteristics at baseline

Characteristic		Total (N = 376) Median (25%,75%) or mea ± SD	
Age		60 (53, 65)	
Sex	Female	158 (42%)	
	Male	218 (58%)	
BMI		23.86 ± 3.2	
Address	Country	155 (41.2%)	
	Town	221 (58.8%)	
Smoking status	Former	164 (43.6%)	
	Never	43.6 (56.4%)	
Tumor site	RUL	81 (21.5%)	
	LUL	103 (27.4%)	
	RLL	82 (21.8%)	
	LLL	83 (22.1%)	
	RML	27 (7.2%)	
Histologic subtype	Adeno	233 (62%)	
	Squamous	90 (23.9%)	
	SCLC	25 (6.6%)	
	Another	28 (7.4%)	
Differentiation	Well	66 (17.6%)	
	Moderately	90 (23.9%)	
	Poorly	220 (58.5%)	
PIS	Yes	94 (25%)	
	No	282 (75%)	
TNM staging	I	205 (54.5%)	
	II	171 (45.5%)	
ECOG PS	0–1	320 (85.1%)	
	2–4	56 (14.9%)	
Basic illness	Hypertension	59 (15.7%)	
	Diabetes	20 (5.3%)	
Tumor size		32.68 ± 16.62	
Lung function	FEV1	2.37 ± 0.63	
	FEV1%	78.65 ± 9.84	
	DLCO	6.74 ± 1.91	

BMI, body mass index; ECOG PS, Eastern Cooperative Oncology Group performance status; TNM staging, tumor, nodes, metastasis-classification staging; PIS: Pleural invasion status; HOM: History of malignancy; RUL: Right upper lobe; RML: Right middle lobe; RLL: Right lower lobe; LUL: Left upper lobe; LLL: Left lower lobe;

We identified four preoperative peripheral blood biomarkers by referring to relevant studies based on their clinical significance [9, 10]. Some of the peripheral blood biomarker data for all patients are shown in Table 2. Based on the best CUT-OFF, low MLR (239/376, 63,6%), low NLR (169/376, 44.9%), low PLR (209/376, 55.6%) and low D-dimer (113/376, 30.3%) were predominant. The results for best CUT-OFF values are found in the Additional file 1

**Table 2:** Patient's peripheral blood data

Characteristic	Median (25%,75%)	Total (n = 376)	
		High (%)	low (%)
Neutrophil count	4.07 (3.13, 5.04) (10^9/L)		
Lymphocyte count	1.87 (1.5, 2.34) (10^9/L)		
Monocyte count	0.36 (0.25, 0.46) (10^9/L)		
Platelet count	237 (196, 285) (10^9/L)		
D-Dimer	110.5 (62.25, 168) (ng/ml)	263 (69.7%)	113 (30.3%)
MLR	0.22 (0.14, 0.25)	137 (36.4%)	239 (63.6%)
NLR	2.61 (1.58, 2.94)	207 (55.1%)	169 (44.9%)
PLR	137.6 (96.73, 162.73)	167 (44.4%)	209 (55.6%)

MLR, monocyte to lymphocyte ratio; NLR, neutrophil to lymphocyte ratio; PLR, platelet to lymphocyte ratio

### Univariate and multivariate cox analysis for survival outcome

**Fig. 1:**
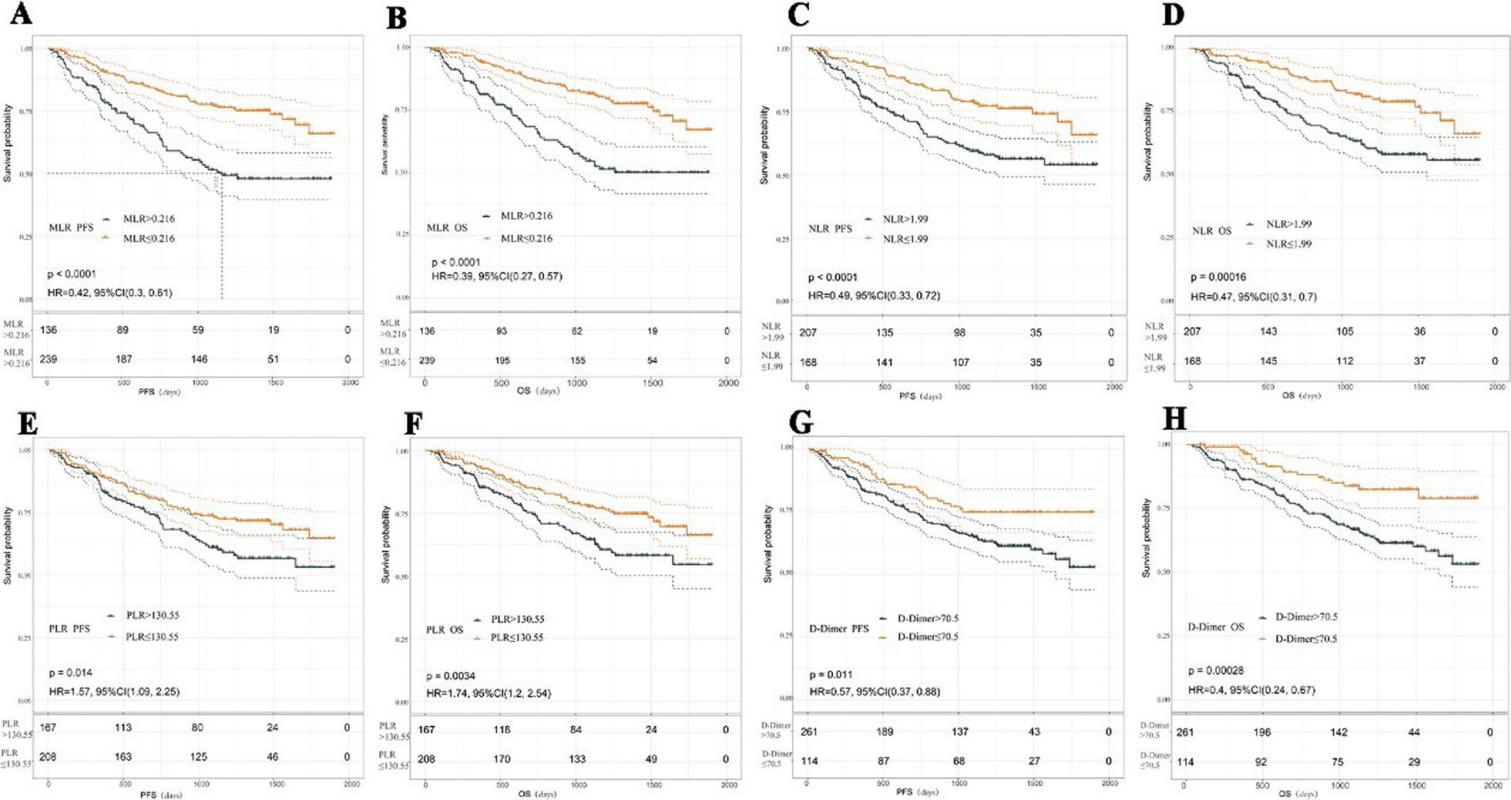
PFS (**A**, **C**, **E**, **G**) and OS (**B**, **D**, **F**, **H**) curves of patients stratified according to peripheral blood markers (MLR, NLR, PLR and D-dimer). *p *values were calculated with the log-rank test.

The results of OS and PFS based on peripheral blood cell counts are shown in Fig. 1: pretreatment low NLR (log-rank test, *p* <0.001), low NLR (*p* <0.001), low PLR (*p* = 0.003) and low D-dimer (*p* <0.001)). We performed a univariate Cox analysis including 4 kinds of preoperative peripheral blood data and the basic clinical information of patients. The results showed that female sex, lack of smoking, SCLC and squamous cell carcinoma, low MLR, low PLR, low NLR, low D-dimer, and ECOG PS (0–1) were associated with significantly better OS and PFS (all *p* <0.05) (Table 3).

**Table3:** Univariate analyses of biomarkers for OS and PFS

Characteristic	Reference	OS			PFS		
		HR	95% CI	*p *value	HR	95% CI	*p *value
Sex	Male	2.44	1.59–3.75	< 0.001	2.26	1.51–3.40	< 0.001
Age		1.01	0.99–1.03	0.241	1.01	0.99–1.03	0.257
Smoking	Yes	1.69	1.16–2.46	0.007	1.53	1.07–2.20	0.021
Pathology	Adeno						
	Another	1.73	0.85–3.51	0.128	1.7	0.87–3.32	0.122
	SCLC	2.86	1.49–5.47	0.002	2.53	1.33–4.81	0.005
	Squama	1.96	1.29–3.00	0.002	1.82	1.21–2.75	0.004
Size		1.01	0.99–1.02	0.327	1.01	1.00–1.02	0.262
PIS	Yes	1.78	1.20–2.64	0.004	1.96	1.35–2.85	< 0.001
TNM	II	1.25	0.86–1.83	0.238	1.32	0.92–1.89	0.133
BMI		0.97	0.92–1.03	0.369	0.98	0.93–1.04	0.527
MLR	Low	0.39	0.27–0.57	< 0.001	0.43	0.30–0.61	< 0.001
NLR	Low	0.46	0.31–0.70	< 0.001	0.49	0.33–0.72	< 0.001
PLR	Low	0.57	0.39–0.83	0.004	0.64	0.44–0.91	0.014
D-Dimer	Low	0.4	0.24–0.67	< 0.001	0.57	0.37–0.89	0.013
ECGO PS	0–1	0.26	0.18–0.39	< 0.001	0.21	0.14–0.31	< 0.001

Given that variables identified as affecting outcome by univariable analysis might be covariates, we performed multivariable Cox proportional regression analysis with parameters found to have a P value less than 0.05 in the univariable analysis to identify independent factors related to the efficacy of surgery in terms of PFS and OS. The results showed that the preoperative peripheral blood biomarkers MLR and D-dimer (*p* =  0.009, 0.005, respectively), which we observed, maintained their significance for OS. However, D-dimer was not significant for PFS (*p* =  0.077) (Table 4).

**Table 4:** Multivariate analyses of biomarkers for OS and PFS

Characteristic	Reference	OS			PFS		
		HR	95% CI	*p *value	HR	95% CI	*p *value
Sex	male	1.72	1.06–2.80	0.0284	1.51	0.96–2.39	0.0773
Pathology	Adeno						
	Another	1.41	0.69–2.90	0.3449	1.47	0.75–2.91	0.2652
	SCLC	2.25	1.14–4.44	0.0189	2.44	1.26–4.72	0.0083
	Squama	1.56	0.98–2.46	0.0582	1.61	1.03–2.51	0.0379
PIS	yes	1.48	0.97–2.28	0.0697	1.61	1.07–2.40	0.0218
MLR	low	0.56	0.37–0.87	0.0092	0.57	0.39–0.85	0.0055
PLR	low	0.74	0.49–1.12	0.1575			
D-Dimer	low	0.48	0.29–0.80	0.0054	0.67	0.43–1.04	0.0766
ECGO PS	0–1	0.28	0.18–0.42	< 0.0001	0.23	0.15–0.34	< 0.0001

### Multivariate model for survival of patients

**Fig. 2:**
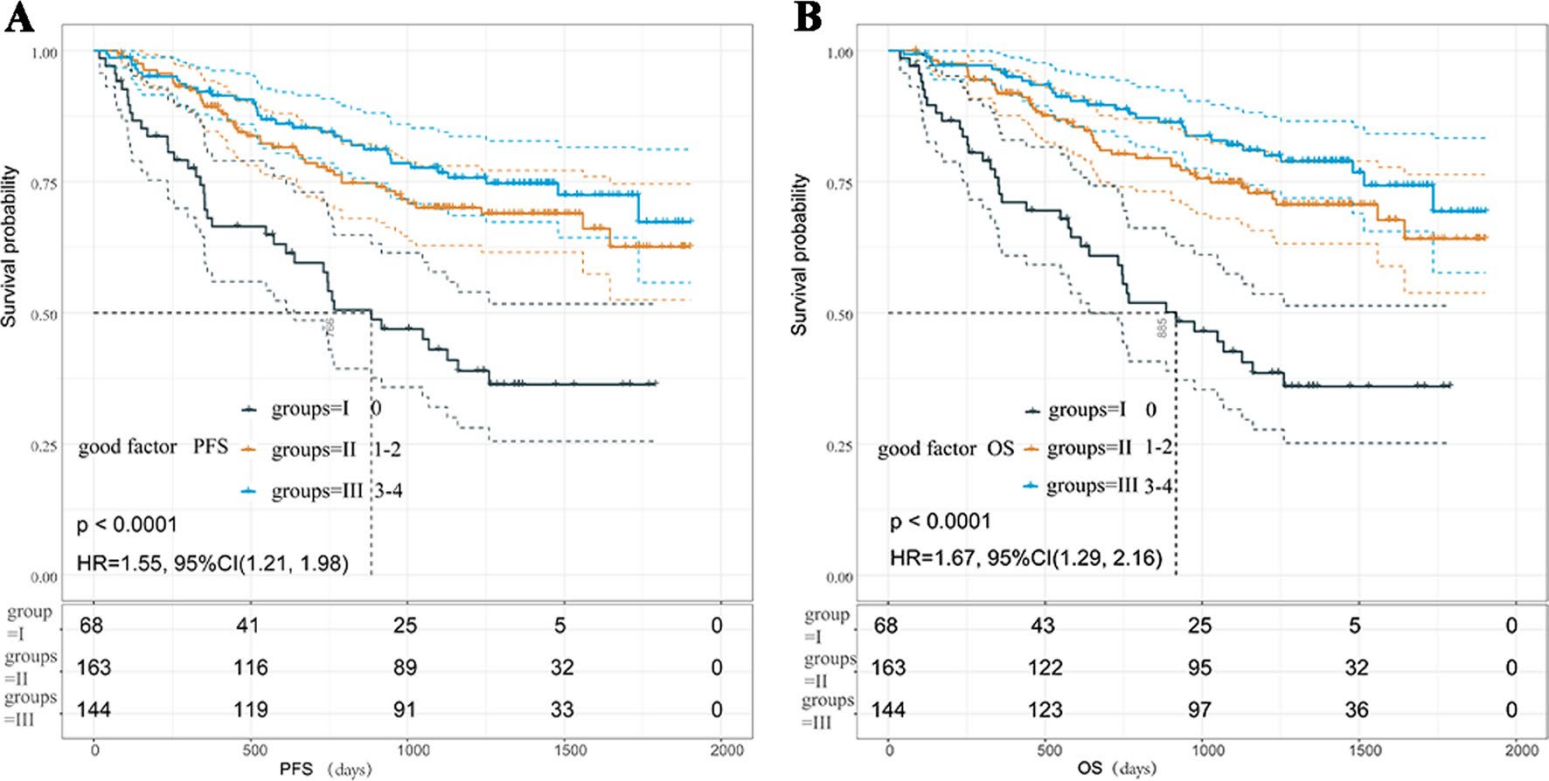
The PFS (**A**) and OS (**B**) were determined for patients in Groups I, II, and III (none, one and two, three and four factors, respectively). *p* values were calculated with the log-rank test

According to the results of the quantitative analysis, the favourable factors included low MLR, low NLR, low PLR and low D-dimer. As shown in Fig. 2, sixty-eight patients (18%) showed no significant reductions in PFS and OS was observed relative to those of patients who had one and two (group II, at 71 and 93, respectively) or three and four (group III, at 102 and 42, respectively) (Kaplan–Meier analysis and survival rates compared to *p* <0.001, respectively). Multivariate Cox analysis including clinically important covariates (age, sex, BMI, ECOG ps, etc.) confirmed that the number of favourable factors was closely associated with PFS and OS (Table 5).

**Table 5:** Multivariate COX regression analysis of OS and PFS grouped by beneficial factors

Group	Total (n = 376)	OS			PFS		
		HR	95% CI	*p *value	HR	95% CI	*p *value
I(0)	68	Reference			Reference		
II(1–2)	164	0.40	0.26–0.62	< 0.0001	0.48	0.31–0.74	0.0008
III(3–4)	144	0.29	0.17–0.48	< 0.0001	0.37	0.23–0.59	< 0.0001

Covariables included age, Eastern Cooperative Oncology Group performance status (0–1 or 2–4), smoking status (former or never).I grope: with no beneficial factors, II grope: There are 1–2 beneficial factors, III grope: There are 3–4 beneficial factors

## Discussion

To date, biomarkers remain a major focus of research in the field of oncology for the diagnosis of disease in patients, the assessment of the efficacy of treatment, and the prognosis of patients. The connection between inflammation and cancer was first explored in 1863 by Rudolf Virchow et al. [11]. Since then, an increasing number of studies have further confirmed the value of inflammatory markers in the diagnostic and prognostic evaluations of patients with various malignant tumours.

Various studies have shown that NLR, PLR, MLR and D-dimer are closely related to the inflammation and immune status of cancer patients and have applied them to predict the prognosis of patients with various solid tumours [4–7]. The aim of this study was to identify baseline peripheral blood biomarkers associated with clinical outcome in patients with early lung cancer (stage I-II) treated with surgery. We screened a total of 376 patients with early-stage lung cancer who underwent standard lobectomy. The median follow-up times for PFS and OS in our study were 1090.5 days and 1110 days, respectively. During the median follow-up time, 107 patients progressed, and 94 patients died. Among them, 84 (84/376, 22.34%) patients received postoperative adjuvant therapy, including chemotherapy (58 cases), immunotherapy (17 cases), and targeted therapy (9 cases). To explain whether postoperative adjuvant medication would interfere with our findings, we performed a Kaplan–Meier analysis of PFS and OS in all patients who received postoperative adjuvant medication according to the best CUT-OFF for blood biomarkers, while the log-rank test was used for comparison (in Additional file 3). The results showed that the OS and PFS of patients receiving adjuvant therapy were not significantly correlated with the MLR, PLR and D-dimer metrics. However, there was a significant difference in NLR, which we think may be due to selection bias (Additional file 3). Some studies show that the albumin to fibrinogen ratio (AFR) could be used to predict the clinical efficacy of chemo‐radiotherapy, combined surgical resection, and chemo‐radiotherapy treatment and could improve the prognosis of low AFR stage II–III patients [12].

Inflammatory mediators in the tumour microenvironment play a major role in the body's immune surveillance and serve as a protective prognostic factor for patients with malignancies [13–15]^.^ Although the mechanism of plasma D-dimer function in tumour development is still unclear, some studies have reported that elevated plasma D-dimer levels in breast cancer patients are associated with progesterone receptor expression, TNM staging and metastasis in breast cancer [16]. Our research finally showed that MLR and D-dimer can also be proposed as independent predictive markers of prognosis in patients with surgically treated lung cancer. In addition, we also found that ECOG ps (0–1) was associated with significantly better OS (HR = 0.26, 95% CI 0.18–0.39, *p* <0.001) and PFS (HR = 0.21, 95% CI 0.14–0.31, *p* <0.001). However, a trend towards worse PFS and OS was apparent in patients who were male and who smoked. Furthermore, we are not aware of any studies that have examined whether there is superposition between these markers or whether there is an interaction between them. For this reason, we divided these markers into three groups according to the number of beneficial factors, and the results of a multifactorial Cox regression showed that patients in the group without a single beneficial factor had worse prognoses than those of the other two groups, while patients in the group with the most beneficial factors had the best prognoses. It is reasonable to believe that a number of the beneficial peripheral blood biomarkers described above could be used as valid predictors of the prognostic status of patients. Based on the above research, we hope to intervene in in the disease progression of patients who may have poor survival through the use of the preoperative blood biomarkers we screened to prolong the survival time of such patients.

## Conclusion

A baseline signature of a low MLR, low NLR, low PLR, and low D-dimer was associated with a better patient survival following surgerical treatment. Patients with more beneficial factors have better survival outcomes.

## Supplementary Information

**Additional file 1**. CUT-OFF values for MLR, NLR, PLR and D-Dimer.

**Additional file 2.** Clinical baseline data between high and low MLR, NLR, PLR and D-Dimer groups.

**Additional file 3.** After postoperative adjuvant medication,relationship between high and low groups of MLR, NLR, PLR and D-Dimer and survival time of patients.

## Data Availability

The datasets generated and/or analysed during the current study are not publicly available due [Our ethics committee stipulates that the information that can identify patients will not be disclosed to members other than the research team] but are available from the corresponding author on reasonable request.

## Abbreviations

MLR: Monocyte-to-lymphocyte ratio
NLR: Neutrophil-to-lymphocyte ratio
PLR: Platura-to-lymphocyte ratio
D-dimer: Dimeric fibrin fragment D
LC: Lung Cancer
PFS: Progression-free survival
OS: Overall survival
TNM: Tumor node metastases
NSCLC: Non-small-cell lung cancer
ROC: Receiver operating Characteristic
CI: Confidence interval
HR: Risk ratio
CTL: Cytotoxic T lymphocytes
AFR: Albumin to fibrinogen ratio
PIS: Pleural invasion status
