# Supplementary material for: The MLR, NLR, PLR and D-dimer are associated with clinical outcome in lung cancer patients treated with surgery
Source: BMC Pulm Med. 2022 Mar 25;22:104. doi: 10.1186/s12890-022-01901-7 (PMC8957174; doi:10.1186/s12890-022-01901-7)
Supplement: Supplementary file 1 — Additional file 1. CUT-OFF values for MLR, NLR, PLR and D-Dimer. [file 12890_2022_1901_MOESM1_ESM.docx]

**
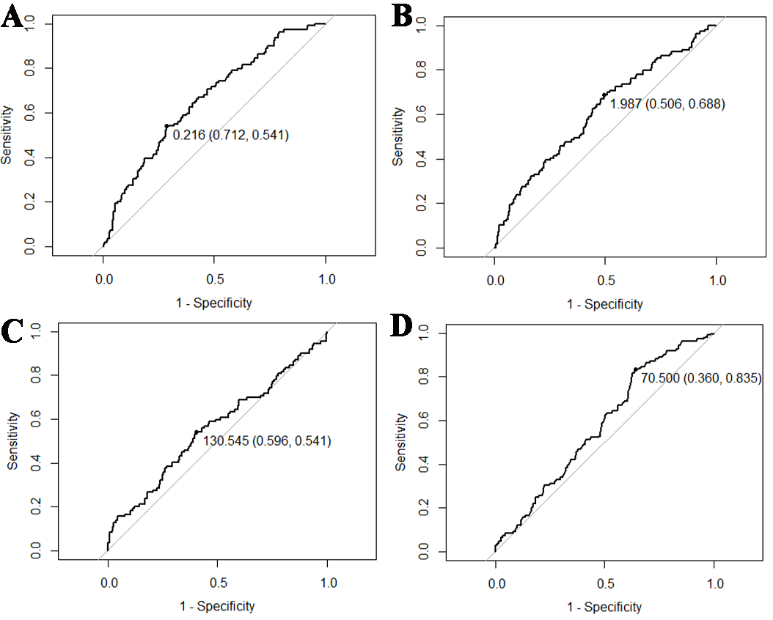
Supplementary material 1**

Receiver operating characteristic (ROC) curves were generated to search for the best cut-off values. Figures A, B, C and D are the best CUT-OFF values of MLR, NLR, PLR and D-Dimer, respectively
