# Supplementary material for: The MLR, NLR, PLR and D-dimer are associated with clinical outcome in lung cancer patients treated with surgery
Source: BMC Pulm Med. 2022 Mar 25;22:104. doi: 10.1186/s12890-022-01901-7 (PMC8957174; doi:10.1186/s12890-022-01901-7)
Supplement: Supplementary file 2 — Additional file 2. Clinical baseline data between high and low MLR, NLR, PLR and D-Dimer groups. [file 12890_2022_1901_MOESM2_ESM.docx]

**Table 1** **Clinical characteristics of patients grouped by MLR level**

| Characteristic | | MLR | | X^2^ | P |
| --- | --- | --- | --- | --- | --- |
|  |  | **low** | **high** |  |  |
| Sex | Female  Male | 131  108 | 27  110 | 44.045 | ＜0.001 |
| Address | Country  Town | 88  151 | 67  70 | 5.249 | 0.023 |
| Smoking status | Former  Never | 86  153 | 78  59 | 15.543 | ＜0.001 |
| Tumor site | RUL  LUL  RLL  LLL  RML | 47  73  52  51  16 | 34  30  30  32  11 | 3.827 | 0.432 |
| Histologic subtype | Adeno.  Squamous.  SCLC  Another | 163  45  15  16 | 70  45  10  12 | 11.897 | 0.007 |
| Differentiation | Well  Moderately  Poorly | 47  62  130 | 19  28  90 | 4.669 | 0.097 |
| PIS | Yes  No | 55  184 | 39  98 | 1.382 | 0.266 |
| TNM staging | I  II | 129  110 | 76  61 | 0.079 | 0.830 |
| Basic illness | Hypertension  Diabetes | 34  13 | 25  7 | 1.065  0.019 | 0.306  0.891 |
| ECOG PS | 0-1  2-4 | 207  32 | 113  24 | 1.171 | 0.294 |
| NLR | Low  High | 152  87 | 17  120 | 92.217 | ＜0.001 |
| PLR | Low  High | 155  54 | 84  83 | 22.824 | ＜0.001 |
| D-Dimer | Low  High | 83  156 | 31  106 | 6.035 | 0.015 |

**Table 2** **Clinical characteristics of patients grouped by NLR level**

| Characteristic | | NLR | | X^2^ | P |
| --- | --- | --- | --- | --- | --- |
|  |  | **low** | **high** |  |  |
| Sex | Female  Male | 92  77 | 66  141 | 19.425 | ＜0.001 |
| Address | Country  Town | 63  106 | 92  115 | 1.972 | 0.172 |
| Smoking status | Former  Never | 66  103 | 98  109 | 2.600 | 0.117 |
| Tumor site | RUL  LUL  RLL  LLL  RML | 32  50  36  37  14 | 49  53  46  46  13 | 2.068 | 0.725 |
| Histologic subtype | Adeno.  Squamous.  SCLC  Another | 116  35  8  10 | 117  55  17  18 | 6.197 | 0.102 |
| Differentiation | Well  Moderately  Poorly | 33  47  89 | 33  43  131 | 4.400 | 0.111 |
| PIS | Yes  No | 34  135 | 60  147 | 3.902 | 0.056 |
| TNM staging | I  II | 101  68 | 104  103 | 3.402 | 0.077 |
| Basic illness | Hypertension  Diabetes | 20  9 | 39  11 | 3.452  0.000 | 0.066  1 |
| ECOG PS | 0-1  2-4 | 146  23 | 174  33 | 0.399 | 0.563 |
| MLR | Low  High | 152  17 | 87  120 | 92.127 | ＜0.001 |
| PLR | Low  High | 38  131 | 78  129 | 59.797 | ＜0.001 |
| D-Dimer | Low  High | 57  112 | 57  150 | 1.688 | 0.215 |

**Table 3** **Clinical characteristics of patients grouped by PLR level**

| Characteristic | | PLR | | X^2^ | P |
| --- | --- | --- | --- | --- | --- |
|  |  | **low** | **high** |  |  |
| Sex | Female  Male | 82  127 | 76  91 | 1.500 | 0.248 |
| Address | Country  Town | 85  124 | 70  97 | 0.060 | 0.833 |
| Smoking status | Former  Never | 98  111 | 66  101 | 2.050 | 0.174 |
| Tumor site | RUL  LUL  RLL  LLL  RML | 42  59  44  49  15 | 39  44  38  34  12 | 1.101 | 0.894 |
| Histologic subtype | Adeno.  Squamous.  SCLC  Another | 129  56  10  14 | 104  34  15  14 | 4.424 | 0.220 |
| Differentiation | Well  Moderately  Poorly | 40  54  115 | 26  36  105 | 2.362 | 0.307 |
| PIS | Yes  No | 47  162 | 47  120 | 1.584 | 0.231 |
| TNM staging | I  II | 110  99 | 95  72 | 0.678 | 0.466 |
| Basic illness | Hypertension  Diabetes | 27  13 | 32  7 | 2.735  0.758 | 0.117  0.490 |
| ECOG PS | 0-1  2-4 | 179  30 | 141  26 | 0.108 | 0.772 |
| NLR | Low  High | 131  78 | 38  129 | 59.797 | ＜0.001 |
| MLR | Low  High | 155  54 | 84  83 | 22.824 | ＜0.001 |
| D-Dimer | Low  High | 73  136 | 41  126 | 4.732 | 0.032 |

**Table 4 Clinical characteristics of patients grouped by D-Dimer level**

| Characteristic | | D-Dimer | | X^2^ | P |
| --- | --- | --- | --- | --- | --- |
|  |  | **low** | **high** |  |  |
| Sex | Female  Male | 49  65 | 109  153 | 0.062 | 0.821 |
| Address | Country  Town | 48  66 | 107  155 | 0.053 | 0.821 |
| Smoking status | Former  Never | 44  70 | 120  142 | 1.677 | 0.214 |
| Tumor site | RUL  LUL  RLL  LLL  RML | 29  31  23  23  8 | 52  72  59  60  19 | 1.629 | 0.804 |
| Histologic subtype | Adeno.  Squamous.  SCLC  Another | 78  24  2  10 | 155  66  23  18 | 7.948 | 0.047 |
| Differentiation | Well  Moderately  Poorly | 20  32  62 | 46  58  159 | 1.644 | 0.443 |
| PIS | Yes  No | 27  87 | 67  195 | 0.151 | 0.796 |
| TNM staging | I  II | 58  56 | 147  115 | 0.876 | 0.369 |
| Basic illness | Hypertension  Diabetes | 13  9 | 46  11 | 2.274  2.155 | 0.165  0.209 |
| ECOG PS | 0-1  2-4 | 98  16 | 222  40 | 0.095 | 0.875 |
| NLR | Low  High | 57  57 | 112  150 | 1.688 | 0.215 |
| PLR | Low  High | 73  41 | 136  126 | 4.732 | 0.032 |
| MLR | Low  High | 83  31 | 156  106 | 6.035 | 0.015 |

**Table 5. median follow-up time**

|  | median follow-up time /Med (P_25_,P_75_) | Number of cases during median  follow-up time（cases） |
| --- | --- | --- |
| OS（d） | 1110（550，1441.75） | 94（25%） |
| PFS（d） | 1090.5（457.75，1436.25） | 107（28%） |

**Table 6.** **Blood Cell Biomarker Characteristics**

| blood cell biomarkers |  | Number of observed outcomes | | X^2^值 | P值 |
| --- | --- | --- | --- | --- | --- |
|  |  | low | high |  |  |
| MLR | PFS  OS | 56  50 | 62  59 | 19.262  20.746 | ＜0.001  ＜0.001 |
| NLR | PFS  OS | 38  34 | 80  75 | 11.286  11.735 | ＜0.001  ＜0.001 |
| PLR | PFS  OS | 56  50 | 62  59 | 4.601  5.866 | 0.034  0.017 |
| D-Dimer | PFS  OS | 25  18 | 93  91 | 6.789  13.847 | 0.011  ＜0.001 |
