# Supplementary material for: The MLR, NLR, PLR and D-dimer are associated with clinical outcome in lung cancer patients treated with surgery
Source: BMC Pulm Med. 2022 Mar 25;22:104. doi: 10.1186/s12890-022-01901-7 (PMC8957174; doi:10.1186/s12890-022-01901-7)
Supplement: Supplementary file 3 — Additional file 3. After postoperative adjuvant medication,relationship between high and low groups of MLR, NLR, PLR and D-Dimer and survival time of patients. [file 12890_2022_1901_MOESM3_ESM.docx]

**Supplementary material 3
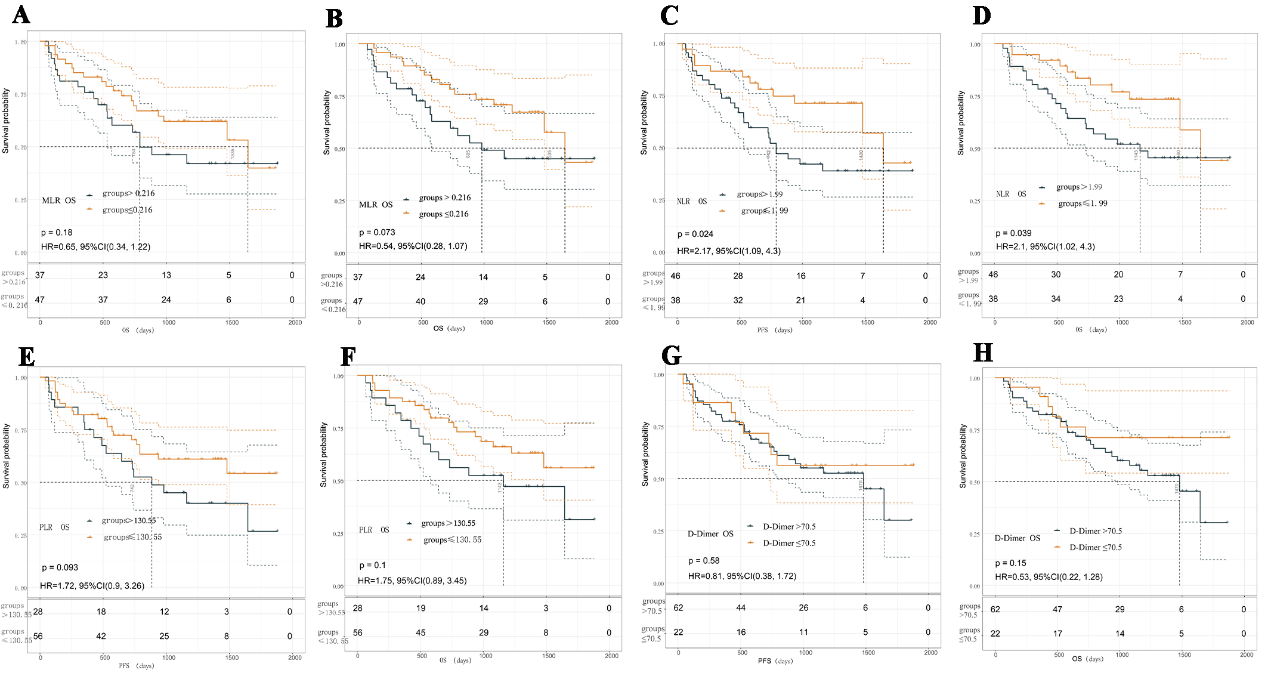
**

To explain whether postoperative adjuvant medication would interfere with our findings, we performed a Kaplan–Meier analysis of PFS and OS in 84 patients who received postoperative adjuvant medication according to the best CUT-OFF for blood biomarkers, while the log-rank test was used for comparison (in supplementary material). PFS (A, C, E, G) and OS (B, D, F, H) curves of patients stratified according to peripheral blood markers (MLR, NLR, PLR and D-dimer)
